# Report of a Case of Coup De Soleil, with Post-mortem Examination, and Remarks

**Published:** 1860-09

**Authors:** Humphrey Peake

**Affiliations:** Yazoo City, Mississippi


ART. III.—*Report of a Case of* Coup de Soleil, *with Post-mortem Examination and Remarks.* By HUMPHREY PEAKE, M.D., of Yazoo City, Mississippi.

ON Thursday morning, July twenty-first, I was sent for to visit a negro man fifteen miles in the country, and arriving at half-past ten o'clock, was furnished with the following history:—

The patient, a stout negro man, of the age of about thirty years, has been a runaway for near six weeks. He was caught a few days ago and put to work in the field. The weather for the past three days has been intensely hot, the mercury having marked on Fahrenheit's scale a temperature of from 96° to 98° in the shade during the heat of the day. Late in the afternoon of yesterday, this man, while at work in the field with other hands, fell down and was carried to the house. He did not go to his dinner, but spent the time allowed for this purpose in an orchard. For the past three or four days he has been working bareheaded. He never spoke or gave any evidence of intelligence after falling, neither could he swallow. After having been carried to the house he was cupped by his master freely in the regions of the temples and on the back of the neck. He was also put in a hot mustard-bath, and afterwards had sinapisms applied to his legs and wrists. During the night he vomited an enormous quantity of half-chewed apples, which seemed not to have been affected in the least by the action of the stomach. It was observed that his skin was intensely hot, that he passed fæces and urine involuntarily, and that he seemed not to have any use of himself whatever.

Every expiration is attended with a kind of half groan and half sigh; tracheal râles are heard at the distance of several yards; the chest expands but very little, and the breathing is almost entirely diaphragmatic; the pomum Adami moves violently up and down with inspiration and expiration; the under jaw is considerably fallen, and a white froth is issuing from the mouth; a perfect tornado of râles and rhonchi are heard on auscultating the chest, and these entirely mask the sounds of the heart. On applying the hand to the chest, a sensation is communicated as of fluids boiling and bubbling beneath; the pulse is beating one hundred and sixty times in the minute, and is irregular in force as well as in rhythm; the eyes are open, and present that dry, glazed or horny appearance which the observant physician has not failed to notice sometimes in the moribund. I have no means of determining the temperature of the skin, but it literally burns my hand; it can be distinctly appreciated by holding the hand two feet from the body.

I informed the master of my opinion of the nature of the case, at the same time telling him that I thought nothing could be done. As a forlorn hope, however, and as fulfilling the most prominent indication, I proposed pouring a quantity of cold water upon him, taking care to explain that in all human probability he could live but a very short time, and that it was even probable that he would die under the affusion. This, indeed, turned out to be the case. I had thrown a couple of bucketfuls upon him by handfuls, when the breathing suddenly stopped. Fully a minute after he had drawn the last breath, he drew up his left lower extremity to a semiflexed position, curved his body considerably to the left side, and passed a quantity of fæces.

The post-mortem examination was held half an hour after death. On laying open the chest, near a pint of what seemed to be almost pure blood was found in each pleural cavity; the lungs, particularly the middle portions of the upper lobes, were intensely congested, and their tissue resembled more that of a hypertrophied spleen than healthy lung; a small portion of the lower lobes presented a normal appearance. After their examination, I wondered that the man could have survived so long. The heart seemed healthy, but the pericardial sac contained four or five ounces of a pale serum; the mucous membrane lining the great curvature of the stomach was much congested, and patches of it had a sodden appearance; the spleen was considerably enlarged. The brain was not examined for want of proper utensils, and also for the reason that a sufficient cause to produce death had already been discovered in the lungs. During the examination, I made an opening in one of the jugular veins; a jet of blood flowed from it with considerable force for some time, and formed an arc the chord of which would have measured three or four inches.

*Remarks.*—This, in my opinion, was a case of coup de soleil, better named by Dr. Bennet Dowler, of New Orleans, solar asphyxia. For the opinions of this eminent and observing physician concerning the malady, I beg leave to refer the reader to volume xii. page 474, of the *New Orleans Medical and Surgical Journal.* Who first advanced the idea that death, in cases of coup de soleil, was a consequence of an "expansion of the fluids," I am unable to say, but the investigations of Dr. Dowler have amply corroborated that opinion, and it remained for him to point out the fact that the effects of this expansion were to be sought mainly in the lungs and not in the brain, as has been, and is still generally supposed. No one, perhaps, has so laboriously investigated the symptoms and pathology of this disease as Dr. Dowler, and he has recorded this opinion: "Be it what it may, the cause of death begins, continues, and ends in the breathing apparatus."

That solar heat is the cause of coup de soleil we cannot doubt. In what manner this best operates other than by expanding the fluids, if in any, it is impossible for me to say. Neither can I determine whether the same effects would follow were the temperature of the body increased by other means, to the degree found in sun-stroke. That the "expansion of the fluids," aside from any other influence, should be sufficient to produce death, seems wholly probable. The quantity of blood in the body of an ordinary sized man may be roughly stated at thirty pounds. Now suppose the temperature of this quantity of fluid to be increased ten or fifteen degrees, which is evidently the case in the affection under consideration, in accordance with a well-known law of physics it will undergo a change by which it will be made to occupy more space. The blood in the animal body may be said to occupy a closed and elastic sac, and it may undergo some increase of bulk without the sac's being ruptured. If at any part the walls of this sac be thinner and less able to resist the pressure of the expansive force than another, an accumulation will take place at the weak point—the point of "least resistance."

A consideration of these propositions will enable the reader to understand why in some cases a blood-vessel *may* be found ruptured in the brain in cases of sun-stroke, and why, in the great majority of cases, the lesion is found in the lungs, the peculiar anatomical structure of which will furnish him a key to the solution of the phenomena.

Dr. Dowler has called attention to a remarkable phenomenon in cases of death from this disease—to the post-mortem circulation of the blood. He gives one or two cases in which the heart continued to act for an hour or more after death. He has also shown that in all cases of the worst form of coup de soleil, and which is the variety I have been considering, on opening a vein the blood flowed for some time in a jet. This was observed by me in the case of which I have given a report. It can be accounted for by keeping in view the propositions given above, without supposing the blood to circulate. Imagine an india-rubber bag to be inflated with air, or, better, suppose it to be distended with water; make a puncture in the bag, and the water will flow in a jet with a greater or less force, according to the greater or less degree of force employed in distending the bag. Just so in those cases of coup de soleil, the blood being inclosed in a short elastic sac, and expanded by heat, the sac is distended. If a vessel be opened, just as in the case of the india-rubber bag, the blood flows in a jet.

In relation to the prognosis in the worst form of coup de soleil—the solar asphyxia of Dr. Dowler—he has told us, what is no doubt true, that it is as certainly fatal as hanging or drowning. Cases of a milder form, however, which he terms solar exhaustion, recover as a general rule. The symptoms of the two varieties are totally different, with the exception merely of the falling down of the patient in the latter case, under the same circumstances as the victim of solar asphyxia. The latter form usually proves fatal in an hour. Dr. Dowler saw one case in which death did not supervene until fifteen hours after the beginning of attack. The patient, of whose case I furnish a report, survived about sixteen or seventeen hours.

From a consideration of the evident causes of the malady, its symptoms, and other points, a valuable hint may be gleaned, at least, in regard to the prophylaxis. One of the strongest indications is to reduce the great heat of the body which precedes and accompanies the attack.

